# Construção e Validação de Instrumento sobre o uso de Anticoncepcional Hormonal Oral

**DOI:** 10.15649/cuidarte.1970

**Published:** 2021-09-10

**Authors:** Francisco de Assis Viana-dos-Santos, Alice de Sousa-Ventura, Amanda Bastos-de-Castro, Angelina Monteiro-Furtado, Jéssica de Menezes-Nogueira, Jardeliny Corrêa-da-Penha

**Affiliations:** 1 . Universidade Federal do Piauí, Floriano, Piauí, Brasil. E-mail: assissantosf9@gmail.com Universidade Federal do Piauí Universidade Federal do Piauí Floriano Piauí Brazil assissantosf9@gmail.com; 2 . Universidade Federal do Piauí, Floriano, Piauí, Brasil. E-mail: aliceventura07@hotmail.com Universidade Federal do Piauí Universidade Federal do Piauí Floriano Brazil aliceventura07@hotmail.com; 3 . Universidade Federal do Piauí, Floriano, Piauí, Brasil. E-mail: amandabastos.1710@gmail.com Universidade Federal do Piauí Universidade Federal do Piauí Piauí Brazil amandabastos.1710@gmail.com; 4 . Universidade Federal do Piauí, Floriano, Piauí, Brasil. E-mail: angelinamonteiro1@yahoo.com.br Universidade Federal do Piauí Universidade Federal do Piauí Floriano Piauí Brazil angelinamonteiro1@yahoo.com.br; 5 . Universidade Federal do Piauí, Floriano, Piauí, Brasil. E-mail: jessicademenezesn@gmail.com Universidade Federal do Piauí Universidade Federal do Piauí Piauí Brazil : jessicademenezesn@gmail.com; 6 . Universidade Federal do Piauí, Floriano, Piauí, Brasil. E-mail: jardelinypenha@yahoo.com.br Autor de correspondência Universidade Federal do Piauí Universidade Federal do Piauí Floriano Piauí Brazil jardelinypenha@yahoo.com.br

**Keywords:** Conhecimentos, Atitudes e Prática em Saúde, Anticoncepcionais Orais, Saúde Sexual e Reprodutiva, Estudo de Validação., Health Knowledge, Attitudes and Practice, Oral Contraceptives, Sexual and Reproductive Health, Validation Study., Conocimientos, Actitudes y Práctica en Salud, Anticonceptivos Orales, Salud Sexual y Reproductiva, Estudio de Validación.

## Abstract

**Introdução:**

Os anticoncepcionais hormonais orais estão entre os métodos contraceptivos mais utilizados pelas mulheres, quando usado corretamente apresenta grande eficácia, mas para o uso adequado é fundamental que el as tenham conhecimentos e atitudes adequadas sobre eles.

**Objetivo:**

Construir e validar um inquérito para avaliar os conhecimentos, atitudes e prática sobre o uso de anticoncepcional hormonal oral.

**Métodos:**

Estudo metodológico, que aconteceu em duas etapas: construção do inquérito e validação por juízes especialistas. A análise da validade de conteúdo foi realizada por meio do Índice de Validade de Conteúdo(IVC). Osjuízesavaliaramcadaitemindividualmente, com relação à clareza da linguagem, à pertinência prática e à relevância teórica. A pesquisa foi aprovada por Comitê de Ética em Pesquisa.

**Resultados:**

Na revisão integrativa da literatura, 34 publicações foram selecionadas e a análise delas permitiu a identificação das três categorias: conhecimentos, atitudes e prática quanto ao uso de anticoncepcionais hormonais orais, o que levou à construção do inquérito com 34 questões. Quanto ao IVC, grande parte das questões obteve valor superior a 0,8 (80,0%), o qual foi utilizado como parâmetro. Sobre o IVC total por juiz, em 10 (83,3%) deles se observou também valor superior a 0,8. Já o IVC total do inquérito foi de 0,86.

**Conclusões:**

O inquérito construído mostrou-se ser um instrumento válido para se obter um diagnóstico situacional dos níveis de conhecimentos, de atitudes e prática de uma dada população que faz uso de anticoncepcionais orais.

## Abstract (en)

**Introduction:**

Oral hormonal contraceptives are among the contraceptive methods most used by wom- en, when used correctly, it is highly effective, but for proper use, it is essential that they have adequate knowledge and attitudes about them.

**Objective:**

To build and validate an inquiry to assess knowledge, attitudes and practice on or use of oral hormonal contraceptive.

**Methods:**

Methodological study, which took place in two stages: construction of the survey and validation by expert judges. The content validity analysis was performed using the Content Validity Index (CVI). The judges evaluated each item individ- ually, with respect to language clarity, practical relevance and theoretical relevance. The Research Ethics Committee approved the research.

**Results:**

In the integrative literature review, 34 publications were selected and their analysis allowed the identification of the three categories: knowledge, attitudes and practice regarding the use of oral hormonal contraceptives, which led to the construction of the survey with 34 questions. As for the CVI, most of the questions had a value greater than 0.8 (80.0%), which was used as a parameter. Regarding the total CVI per judge, 10 (83.3%) of them also had a value higher than 0.8. The total CVI of the survey was 0.86.

**Conclusions:**

The constructed survey proved to be a valid instrument for obtaining a situational diagnosis of the levels of knowledge, attitudes and practice of a given population that uses oral contraceptives.

## Resumen

**Introducción:**

Anticonceptivos hormonales orales se encuentran entre los métodos anticonceptivos más utilizados por las mujeres, cuando se usan correctamente es altamente efectivo, pero para su uso adecuado es fundamental que tengan los conocimientos y actitudes adecuados.

**Objetivo:**

Construir y validar una encuesta para evaluar conocimientos, actitudes y prácticas con respecto al uso de anticonceptivos hormonales orales.

**Métodos:**

Estudio metodológico, que se desarrolló en dos etapas: construc- ción de la encuesta y validación por jueces expertos. Análisis de validez de contenido se realizó median- te el Índice de Validez de Contenido (IVC). Jueces evaluaron cada ítem individualmente, con respecto a la claridad del lenguaje, relevancia práctica y relevancia teórica. La investigación fue aprobada por el Comité de Ética en Investigación. Resultados: En la revisión integradora de la literatura se seleccionaron 34 publicaciones y su análisis permitió identificar las tres categorías: conocimientos, actitudes y práctica en relación al uso de anticonceptivos hormonales orales, lo que llevó a la construcción de la encuesta con 34 preguntas. La mayoría de las preguntas tuvo un IVC superior a 0,8 (80,0%), que se utilizó como parámetro. En cuanto al IVC total por juez, 10 (83,3%) de ellos también tenían un valor superior a 0,8. El CVI total de la encuesta fue de 0,86.

**Conclusiones:**

La encuesta construida resultó ser un instrumento válido para obtener un diagnóstico situacional de los niveles de conocimientos, actitudes y prácticas de una determinada población que usa anticonceptivos orales.

## Introdução

Os contraceptivos são métodos de planejamento familiar, os quais são utilizados para prevenir umagravideznãodesejadaounãoplanejada, dentreosquaiscitam-seosmétodoscontraceptivos hormonais orais(1). Estes também chamados de pílulas anticoncepcionais, foram legalizados em 1960, nos Estados Unidos, pela primeira vez(2). No Brasil, passaram a ser distribuídos em 1978(3).

Aliado à prevenção de uma gravidez não desejada ou não planejada, os anticoncepcionais hormonais orais proporcionam à mulher o controle sobre seu corpo e sexualidade, de modo a contribuir para o seu crescimento no mercado de trabalho e principalmente para o desenvolvimentodaautonomiaedaindependênciareprodutiva, promovendo, assim, umgrande impacto no comportamento e posição dela na sociedade(4). Esses contraceptivos permitem que as usuárias fiquem mais despreocupadas com a gravidez e tenham maior liberdade sexual, consequentemente, exibindo maior efeito no aumento da frequência das relações sexuais e no número e intensidade dos orgasmos(5).

Em todo o mundo, os anticoncepcionais hormonais orais são utilizados por cerca de 100 milhões de mulheres em todo o mundo(6). Em território brasileiro, em 2015, as mulheres que usavam algum tipo de método contraceptivo chegavam a 79,0%, contra cerca de 51,0%, em 1970(7), sendo os anticoncepcionais hormonais orais um do mais utilizados devido à praticidade, segurança, facilidade de acesso, eficácia e por não interferir na vida sexual(8).

É fundamental destacar que se estima que mais mulheres usarão contraceptivos até 2030, indo dos 758 milhões para 778 milhões(7). Porém, embora se note elevada prevalência de mulheres em uso do método anticoncepcional hormonal oral, o conhecimento sobre este pode não ser suficientemente adequado, o que poderá interferir no uso correto e consequentemente na eficácia.

Sobre isso, pesquisa realizada em Amorinópolis, Goiás, Brasil, com adolescentes do sexo feminino matriculadas no ensino médio, constatou que: 18,6% conheciam o anticoncepcional hormonal oral; 39,5% referiram que este método serve para regular o ciclo menstrual, seguido de 29,6%, que disseram que previne infecções sexualmente transmissíveis (IST); e 30,0% achavam que deve ser iniciado no final do ciclo menstrual. Os achados evidenciam, portanto, alguns conhecimentos errôneos sobre o uso dos contraceptivos orais(9).

Nesse ínterim, e considerando ainda uma das metas dos Objetivos de Desenvolvimento Sustentável (ODS), especificamente do ODS 3 (três), Saúde e Bem-estar, que visa assegurar o acesso universal aos serviços de saúde sexual e reprodutiva, incluindo o planejamento familiar, informação e educação, bem como a integração da saúde reprodutiva em estratégias e programas nacionais(10), é fundamental o desenvolvimento de ações de educação em saúde sobre métodos anticoncepcionais orais para mulheres em idade fértil (de 10 a 49 anos de idade), a fim de que estes sejam usados utilizados corretamente e tenham eficácia.

Mas para a realização de ações de educação em saúde, é primordial que, inicialmente, sejam investigados os conhecimentos, as atitudes e a prática das mulheres acerca dos métodos citados, a fim de se obter subsídios necessários para o desenvolvimento de estratégias educativas diferenciadas e congruentes com as singularidades do grupo em questão.

Com isso, numa tentativa de coletar informações sobre contracepção e comportamento reprodutivo, pode ser desenvolvido um modelo especial de estudo conhecido como conhecimento, atitude e prática (CAP), este visa o desenvolvimento de programas mais apropriados para as necessidades específicas da população estudada(11),(12).

Os estudos CAP pertencem a uma categoria de estudos avaliativos, chamados de avaliação formativa, ou seja, por meio dos quais se obtêm dados de uma parcela populacional específica, visto que permitem identificar possíveis caminhos para a realização de intervenções eficazes. Por sua vez, consistem em um conjunto de questões que possibilitam medir o que a população sabe, pensa e atua frente a um determinado problema(13)-(15).

A metodologia CAP permite reunir informações de forma dinâmica de uma população a partir do seu conhecimento, da sua atitude e da sua prática, quanto a determinado tema, permitindo um preciso diagnóstico(14)-(16), de modo a oferecer resultados que possibilitem planejar, implementar e avaliar o trabalho a ser desempenhado(14).

Assim, questiona-se: a construção de um inquérito será capaz de avaliar os conhecimentos, atitudes e prática das mulheres sobre o uso de anticoncepcional hormonal oral? Logo, a presente pesquisa objetiva construir e validar um inquérito para avaliar os conhecimentos, atitudes e prática sobre o uso de anticoncepcional hormonal oral. A criação desse inquérito permitirá a avaliação dos conhecimentos, atitudes e prática de mulheres em idade fértil sobre esse método, aspecto fundamental para a elaboração de ações direcionadas ao planejamento familiar e reprodutivo delas.

## Materiais e Métodos

Trata-se de um estudo metodológico de elaboração e validação de conteúdo do inquérito Conhecimentos, Atitudes e Prática sobre o Uso de Anticoncepcional Hormonal Oral, o qual foi desenvolvido em duas etapas:1ª etapa - construção do inquérito CAP; e 2ª etapa - validação do inquérito por juízes especialistas, conforme Fluxograma 1.

### Levantamento teórico e construção do inquérito Conhecimentos, Atitudes e Prática sobre o Uso de Anticoncepcional Hormonal Oral

O levantamento de evidências cientificas se deu por meio da realização de uma revisão integrativa da literatura (RIL), a fim de identificar as principais publicações sobre a temática para embasar a formulação do inquérito CAP, a qual foi realizada no período de junho a agosto de 2019. Para a elaboração da presente revisão, foram utilizadas as seguintes etapas: 1- seleção da questão de estudo, 2- estabelecimento dos critérios para a seleção da amostra, 3- busca na literatura, 4- definição das informações a serem extraídas dos estudos selecionados, 5- avaliação dos estudos incluídos na revisão, e 6- interpretação dos resultados(17).

**Fluxograma 1:**
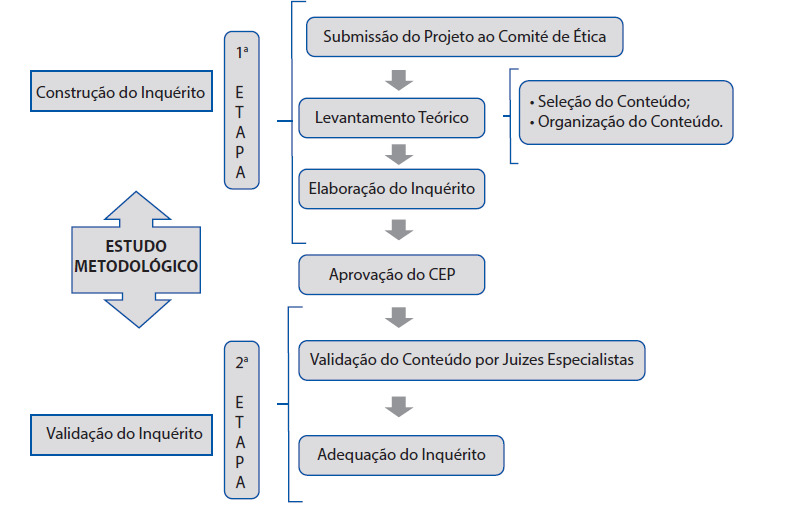
Detalhamento das etapas do estudo. Floriano, Piauí, Brasil, 2020.

Fonte: Dados da Pesquisa, 2020.

A elaboração da questão norteadora constituiu a primeira etapa: quais os conhecimentos, as atitudes e/ou prática das mulheres em idade fértil frente ao uso de contraceptivos/ anticoncepcionais hormonais orais na Atenção Primária à Saúde? Esta questão foi organizada de acordo com a estratégia PICo(18): P - população (mulheres em idade fértil); I − intervenção/ área de interesse (conhecimentos, atitudes e/ou prática frente ao uso de contraceptivos/ anticoncepcionais hormonais orais); Co - contexto (Atenção Primária a Saúde).

Partindo para a segunda etapa, foram adotados como critérios de inclusão: publicações disponíveisgratuitamente, nosidiomasportuguês, inglêseespanhol, depesquisasdesenvolvidas na Atenção Primária à Saúde. Foram excluídos teses, dissertações e editoriais.

Quanto ao levantamento, o qual corresponde à terceira etapa (busca na literatura), foi realizado nos meses de julho a outubro de 2019, via Portal Capes (Coordenação de Aperfeiçoamento de Pessoal de Nível Superior) com acesso às seguintes bases de dados: *Cumulative Index to Nursing and Allied Health Literature* (CINAHL) e *Scopus* (Elsevier). Também foi realizado acesso ao Banco de Dados em Enfermagem (BDEnf), à *Medical Literature Analysis and Retrieval System Online* (Medline), à Literatura Latino-americana e do Caribe em Ciências da Saúde (LILACS), via Biblioteca Virtual em Saúde (BVS).

-Para a busca nas bases de dados, primeiramente foram selecionados descritores em português e, em seguida, buscou-se os equivalentes no banco de descritores da Medical Subject Headings (MeSH) e dos títulos CINAHL. Foram descritores da MeSH: “Women”, “Fertile Period”, “Health Knowledge, Attitudes, Practice”, “Knowledge”, “Attitude”, “Contraceptives, Oral” e “Primary Health Care”, e os títulos CINAHL foram: “Women”, “Knowledge”, “Attitude”, “Contraceptives, Oral” e“Primary Health Care”. Eles foram utilizados por meio de combinações. Destaca-se que a busca na BVS foi realizada utilizando os descritores MeSH.

A partir das combinações inseridas nas bases de dados já citadas e aplicação de critérios de inclusão e exclusão, chegou-se a um total amostral de 34 artigos, conforme apresentado no Fluxograma abaixo (Fluxograma 2).

Após selecionados os estudos que compuseram a amostra, fez-se a extração das informações ou dados desse material. A definição das informações a serem extraídas, equivalente à quarta etapa de uma RIL foi determinada a partir da elaboração de um formulário que continha as seguintes questões: título, ano, base de dados, abordagem, objetivo ou questão de investigação, amostra/sujeitos, resultados e nível de evidência da publicação. Este instrumento de coleta de dados foi adaptado de Ursi(19).

Quanto à análise do material selecionado, ou seja, avaliação dos estudos incluídos na revisão, os achados foram analisados de forma descritiva e crítica, possibilitando observar, contar, descrever e classificar os dados, com o intuito de reunir o conhecimento produzido sobre o tema explorado na revisão(20). Esta análise permitiu identificar os achados que se enquadravam nas categorias previamente determinadas: 1- conhecimentos; 2- atitudes; e 3- prática sobre o uso de contraceptivos hormonais orais.

A partir disso, o inquérito “Conhecimentos, Atitudes e Prática sobre o Uso de Anticoncepcional Hormonal Oral” foi elaborado no período de novembro de 2019 a março de 2020. O primeiro inquérito continha 38 questões, dessas 15 eram referentes aos conhecimentos, 12, às atitudes e as outras 11, à prática. Os próprios pesquisadores avaliaram e refinaram este primeiro inquérito, o que resultou em um instrumento com 34 questões, sendo 15 sobre os conhecimentos, as quais abordavam questões acerca da finalidade da pílula, mecanismo de ação, benefícios não contraceptivos, uso, efeitos colaterais contraindicações e interações medicamentosas; 08, as atitudes, em que estavam presentes perguntas sobre o que ela acha que deve ser o motivos do uso, o que ela acha da forma de usar, e se ela acha que há necessidade de avaliação profissional; e 11, a prática, as quais indagavam sobre motivos do uso, o uso em si e a avaliação por profissional de saúde.

**Fluxograma 2:**
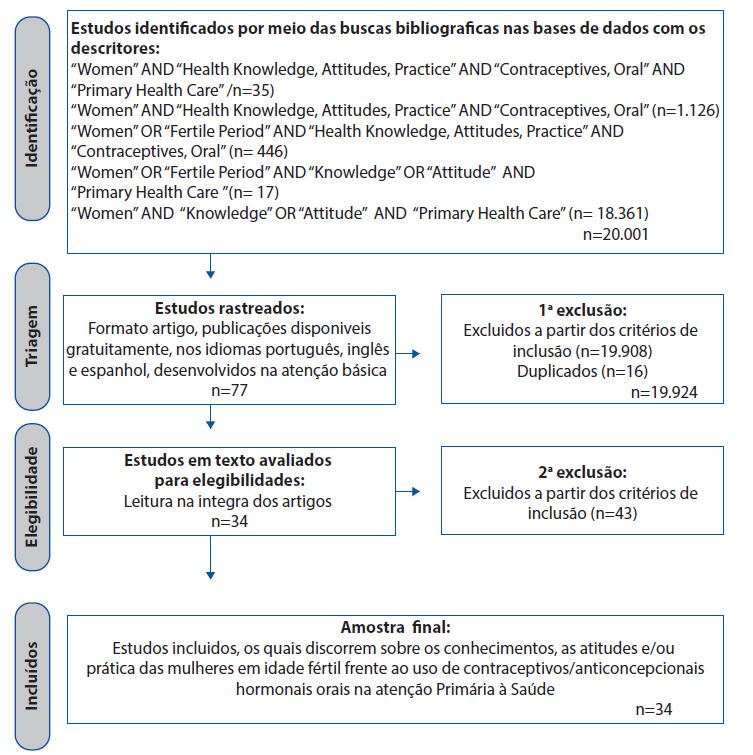
Detalhamento da busca dos artigos nas bases de dados. Floriano, Piauí, Brasil, 2020.

Fonte: Dados da Pesquisa, 2020.

### Validação do inquérito Conhecimentos, Atitudes e Prática sobre o Uso de Anticoncepcional Hormonal Oral por juízes especialistas

O período de validação do inquérito CAP foi de março a agosto de 2020. A seleção dos juízes se deu por conveniência, seguindo o atendimento de critérios de seleção preestabelecidos e adaptados de outros autores(21),(22).

E quanto ao número ideal de juízes para o processo de validação, a literatura é diversificada e não existe uma padronização para tal. Sendo assim, os pesquisadores decidiram por seguir as recomendações de Pasquali(23). Frente a isso, participaram da presente pesquisa 12 juízes especialistas.

Inicialmente, fez-se uma consulta prévia na Plataforma Lattes do portal do Conselho Nacional de Desenvolvimento Científico e Tecnológico (CNPq), a fim de se identificar pesquisadores que contemplassem os critérios estabelecidos. Essa consulta foi realizada mediante a utilização das seguintes combinações: “saúde da mulher e planejamento reprodutivo e conhecimento e atitude e prática”; “saúde da mulher e planejamento reprodutivo e conhecimento e atitude e prática e anticoncepcional oral”; “saúde da mulher e planejamento reprodutivo e validação de inquéritos e conhecimento e atitude e prática”; “saúde da mulher e assistência ao planejamento reprodutivo e validação de inquéritos”; “saúde da mulher e assistência ao planejamento reprodutivo e validação de inquéritos e atitude e prática e anticoncepcional oral”; “saúde da mulher e conhecimento e atitude e prática e anticoncepcional oral”; “anticoncepcional e planejamento reprodutivo e planejamento familiar”. Foram encontrados 158 perfis, desse foram elegíveis para participar da pesquisa 35, para os quais foi enviado o convite.

Além dessa consulta na Plataforma Lattes, houve a indicação pelos pesquisadores de juízes que se enquadravam nos critérios de inclusão, bem como na pontuação mínima exigida, 5 (cinco) pontos.

Para aqueles que aceitaram participar da pesquisa, foi enviado um e-mail, contendo: 1- Termo de Consentimento Livre e Esclarecido (TCLE); 2- questionário de caracterização dos juízes especialistas; 3- instrumento de validação da construção e validação do inquérito; e o 4- inquérito CAP.

O questionário de caracterização dos juízes especialistas continha questões sobre a identificação, qualificação e trajetória profissional. Já o instrumento de validação do inquérito, continha três partes: a primeira, apresentava instruções aos juízes; a segunda, o Quadro de avaliação das 34 questões, por meio de uma escala do tipo Likert (1- pouquíssima, 2- pouca, 3- média, 4- muita e 5- muitíssima), que analisou a clareza da linguagem, a pertinência prática e a relevância teórica; e a terceira, a qual continha uma questão sobre se o Quadro de classificação do conhecimento, da atitude e da prática estava apropriado para determinar se estes itens eram adequados ou inadequados.

Quanto à análise da validade de conteúdo do inquérito, esta foi realizada por meio do Índice de Validade de Conteúdo (IVC). Este índice mede a proporção ou porcentagem de juízes que estão em concordância sobre determinados aspectos do instrumento e de seus itens(24)-(26). Assim, é calculado com a utilização de uma escala do tipo Likert.

O cálculo do IVC, por questões e por juízes, foi feito a partir do somatório das respostas “4” e “5” de cada juiz em cada item do questionário e dividido pelo número total de respostas(24),(27).




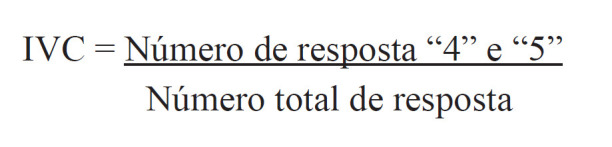




Os itens que receberem pontuação “1” ou “2” ou “3” foram revisados ou eliminados. Destaca-se que foi adotado como valor aceitável de IVC aquele igual ou superior a 0,8 (0,80%)(28),(29), o qual foi adotado em outros estudos de validação(13),(30).

Os dados do IVC foram digitados e analisados no Microsoft Excel, versão 2013. E quanto à caracterização dos juízes, os dados obtidos foram inseridos e analisados no programa estatístico

*Statistical Package for the Social Sciences*, versão 20.0. O tratamento e análise descritiva compreendeu frequências absolutas e relativas e medidas de tendência central (média e mediana).

### Aspectos éticos

A presente investigação seguiu todas as diretrizes brasileiras e internacionais no que concerne à realização de pesquisa envolvendo sendo humanos, de modo a se destacar aprovação dela em Comitê de Ética em Pesquisa (parecer nº 3.563.788). Enfatiza-se ainda que todos os participantes assinaram digitalmente o TCLE, o qual foi devolvido aos pesquisadores. Este documento detalhava claramente a pesquisa, bem como riscos e benefícios, além de informar que não haveria benefício financeiro e que os juízes poderiam desistir da pesquisa a qualquer momento.

## Resultados

### Caracterização dos estudos selecionados para a revisão integrativa da literatura

Inicialmente, apresenta-se uma caracterização dos estudos selecionados na RIL e, conforme se observa no Quadro 1, a base com maior prevalência foi a Medline, com 22 (64,7%) publicações; o ano o mais predominante foi 2014, com 6 (17,6%); a abordagem quantitativa esteve presente na maioria das publicações, em 33 (97,1%); e o nível de evidência prevalente foi o 4, em 26 (76%) publicações.

Não é apresentado no Quadro abaixo, mas se pode constatar que a maioria das pesquisas foram realizadas com mulheres em idade reprodutiva, compreendida entre 18 a 44 anos de idade, e que, em síntese, os estudos objetivaram por pesquisar “a avaliação do conhecimento acerca do uso dos anticoncepcionais”, “o conhecimento das mulheres sobre os riscos e os benefícios dos métodos anticoncepcionais”, “avaliação do uso dos anticoncepcionais pelas mulheres”, “descrever o crenças e comportamentos contraceptivos” e “explorar as opiniões das mulheres sobre uma restrição de idade para o uso das pílulas”.

E em relação aos resultados que responderam à questão norteadora da RIL, eles foram classificar em três categorias: conhecimentos, atitudes e prática quanto uso dos anticoncepcionais hormonais orais. Acerca do conhecimento, identificou-se, em síntese, que as mulheres conhecem os efeitos colaterais, porém há um déficit no que diz respeito ao conhecimento dos efeitos não contraceptivos (1, 3, 23, 31 e 34); elas têm dúvida de como é o funcionamento das pílulas e de como usar (8, 10, 14, 19 e 24); além disso, algumas desconhecem as contraindicações e as interações medicamentosas entre a pílula e outras medicações (15, 25, 28 e 32).

Quanto à atitude, constatou-se que o déficit no conhecimento das usuárias interfere diretamente no modo como elas veem as pílulas, ou seja, o que elas sabem sobre ou não sobre efeitos colaterais, contraindicações e efeitos nãos contraceptivos interferi de modo positivo ou negativo na confiança de las sobre a pílula (3, 4, 5, 6, 9, 11, 13, 17, 20, 27 e 31).

Já com relação à prática, em grande parte, observou-se que a descontinuação está atrelada aos efeitos colaterais menores, em decorrência das mulheres não saberem identificar quais são (04, 05, 0,6, 08 e 32); e a rotina o esquecimento e desabastecimento também são uns dos principais motivos para a descontinuação do uso (22, 27 e 33).

**Quadro 1:** Caracterização dos estudos selecionados para amostra da revisão integrativa da literatura. Floriano, Piauí, Brasil, 2020.

Nº	Título	Base de Dados	Ano	Abordagem	Nível de Evidência
1	Conhecimento de usuárias de anticoncepcional oral combinado de baixa dose sobre o método	LILACS	2013	Quantitativa	04
2	Social, Demographic and Situational Characteristics Assciated with Inconsistent Use of Oral Contraceptives: Evidence from France	CINAHL	2006	Quantitativa	04
3	Womens Knowledge, Beliefs, and Information Needs in Relation to the Risks and Benefits Associated with Use of the Oral Contraceptive Pill	CINAHL	2011	Quantitativa	04
4	The Influence of Oral Contraceptive Knowledge on Oral Contraceptive Continuation Among Young Women	CINAHL	2014	Quantitativa	02
5	Acceptability of the combined oral contraceptive pill among Hong Kong women	Scopus	2016	Quantitativa	04
6	Adherence to the oral contraceptive pill: a cross sectio-nal survey of modifiable behavioural determinants	Scopus	2012	Quantitativa	04
7	Are affluent, well - educated, career- orientated women knowledgeable users of the oral contraceptive pill?	Scopus	2005	Quantitativa	04
8	Current knowledge, attitude, and patterns of oral contraceptives utilization among women in Jordan	Scopus	2015	Quantitativa	04
9	Psychometric Properties of a Measure Assessing Attitudes and Norms as Determinants of Intention to Use Oral Contraceptives	Scopus	2015	Quantitativa	03
10	Relationship between self - efficacy and patient knowledge on adherence to oral contraceptives using the Morisky Medication Adherence Scale (MMAS - 8)	Scopus	2017	Quantitativa	04
11	Young women´s continued use of oral contraceptives over other hormonal methods: findings from a qualitative study	Scopus	2017	Quantitativa	04
12	Misconceptions about the side effects of combined oral contraceptive pills	MEDLINE	2014	Quantitativa	04
13	Impact of a pharmacist - pr-ovided information booklet on knowledge and attitudes towards oral contraception among Jordanian women: na interventional study	MEDLINE	2017	Quantitativa	02
14	Women´s ability to self - screen for contraindications to combined oral contraceptive pills in Tanzanian drug shops	MEDLINE	2013	Quantitativa	04
15	Oral contraceptive use in women at increased risk of breast/ovarian cancer: knowledge and atitudes	MEDLINE	2010	Quantitativa	04
16	Potential public sector cost - savings from over-the- counter access to oral contraceptives	MEDLINE	2015	Quantitativa	04
17	Attitudes Toward Over -the- Counter Access To Oral Contraceptives Among a Sample Of Abortion Clients in the United State	MEDLINE	2014	Quantitativa	04
18	Women´s Perspectives on Age Restrictions for Over -the- Counter Access to Oral Contraceptives	MEDLINE	2015	Quantitativa	04
19	Knowledge and beliefs about reproductive anatomy and physiology among Mexican - Origin women in the USA: implications for effective oral Contraceptive Use	MEDLINE	2013	Quantitativa	04
20	Accuracy of Self - Screening for Contraindications to Combined Oral Contraceptive Use	MEDLINE	2008	Quantitativa	04
21	Young Women's Attitudes Toward Continuous Use of Oral Contraceptives: The Effect of Priming Positive Attitudes Toward Menstruation on Women's Willingness to Suppress Menstruation	MEDLINE	2008	Quantitativa	04
22	Continuous Compared With Cyclic Use of Oral Contraceptive Pills in the Dominican Republic	MEDLINE	2014	Quantitativa	02
23	Perception of oral contraceptives among women of reproductive age in Japan: A comparison with the USA and France	MEDLINE	2011	Quantitativa	04
24	A Survey of Teenagers’ Attitudes Toward Moving Oral Contraceptives Over the Counter	MEDLINE	2015	Quantitativa	04
25	Effect of information on the perception of users and prospective users of combined oral contraceptives regarding the risk of venous thromboembolism	MEDLINE	2014	Quantitativa	04
26	The Relationship Between Acculturation and Oral Contraceptive Use Among Korean Immigrant Women	MEDLINE	2011	Quantitativa	04
27	The Influence of Oral Contraceptive Knowledge on Oral Contraceptive Continuation Among Young Women	MEDLINE	2014	Quantitativa	02
28	Antiepileptic drugs: Are women aware of interactions with oral contraceptives and potential teratogenicity?	MEDLINE	2009	Quantitativa	04
29	The impact of an educational text message intervention on young urban women's knowledge of oral contraception	MEDLINE	2013	Quantitativa	02
30	Oral Contraceptive Use Among Women in the Military and the General U.S. Population	MEDLINE	2010	Quantitativa	02
31	Knowledge matters - Impact of two types of information brochure on contraceptive knowledge, atitudes and intentions	MEDLINE	2012	Quantitativa	02
32	Over -the- counter access to oral contraceptives as a reproductive healthcare strategy	MEDLINE	2013	Revisão	04
33	Combining Qualitative with Quantitative Approaches to Study Contraceptive Pill Use	MEDLINE	1999	Quantitativa	04
34	Oral contraceptive pill use, decisional balance, risk perception and knowledge: An exploratory study	CINAHL	1999	Quantitativa	04

Fonte: Dados da pesquisa, 2020.

### Caracterização dos juízes especialistas e validação de conteúdo

Participaram do estudo 12 (doze) juízes especialistas, com idade entre 23 a 60 anos, com média de 35,58 anos. A maioria deles, 11 (91,7%), era do sexo feminino; com mais de 10 anos de formação, 8 (66,7%); enfermeiro(a), 10 (83,3%); doutor(a), 5 (41,7%); e docente, 7 (58,4%).

O parâmetro utilizado para validar os itens foi de 0,8 (80,0%), ou seja, as questões, com respectivos itens, que obtiveram IVC total menor que 0,8 passaram por um processo de revisão, em que foi decidido pelos pesquisadores se ela seria reformulada ou removida do inquérito. Além disso, foram consideradas também as sugestões dos juízes sobre cada item, mesmo que tivesse um IVC≥0,8. Assim, são apresentados na tabela 1 o ICV de cada item (clareza, pertinência e relevância) e do total por questão.

**Tabela 1:** Apresentação do Índice de Valide de Conteúdo da clareza, pertinência, relevância e total de cada questão do Inquérito Conhecimentos, Atitudes e Prática sobre o Uso do Contraceptivo Oral. Floriano, Piauí, Brasil, 2020.

Inquérito CAP	Clareza	Índice de Validade de Conteúdo				Total
		Pertinência	Relevância	
Questão 1	0,91	0,75	0,75	0,80
Questão 2	0,75	0,83	0,91	0,83
Questão 3	0,50	0,83	0,83	0,72
Questão 4	0,50	0,91	0,91	0,77
Questão 5	0,83	0,91	0,91	0,88
Questão 6	0,91	0,91	0,91	0,91
Questão 7	1,00	0,91	0,91	0,94
Questão 8	0,91	0,91	0,90	0,90
Questão 9	0,91	1,00	1,00	0,97
Questão 10	0,83	0,75	0,83	0,80
Questão 11	0,58	0,83	0,83	0,74
Questão 12	0,58	0,91	0,91	0,80
Questão 13	0,66	0,83	0,91	0,80
Questão 14	0,58	1,00	1,00	0,86
Questão 15	0,75	0,75	0,83	0,77
Questão 16	0,75	0,83	0,83	0,80
Questão 17	0,91	0,83	0,83	0,85
Questão 18	0,91	0,75	0,75	0,80
Questão 19	0,91	0,91	0,83	0,88
Questão 20	0,83	0,75	0,75	0,77
Questão 21	0,75	0,75	0,75	0,75
Questão 22	0,75	0,75	0,75	0,75
Questão 23	0,83	0,91	0,91	0,88
Questão 24	0,91	0,91	0,91	0,91
Questão 25	0,91	0,91	0,91	0,91
Questão 26	0,91	0,91	0,91	0,91
Questão 27	0,91	0,83	0,83	0,85
Questão 28	0,83	0,91	0,91	0,88
Questão 29	1,00	1,00	1,00	1,00
Questão 30	0,83	0,91	0,91	0,88
Questão 31	0,91	1,00	1,00	0,97
Questão 32	0,83	1,00	1,00	0,94
Questão 33	0,91	1,00	1,00	0,97
Questão 34	0,83	1,00	1,00	0,94

Fonte: Dados da pesquisa, 2020.

Como observado na tabela 1, as questões 02, 03, 04, 11, 12, 14, 15, 16, 20, 21 e 22 obtiveram IVC menor que 0,80, portanto, foram obrigatoriamente revisadas ou removida. Já as questões 07, 08, 09, 13, 17, 23, 25, 26, 27, 30, 32 e 34, mesmo com IVC≥0,8, foram verificadas para revisão, considerando as sugestões dos juízes, as quais estão apresentadas no Quadro 2.

**Quadro 2:** Detalhamentos das sugestões dos juízes especialistas e decisão dos pesquisadores por questões do inquérito CAP construído. Floriano, Piauí, Brasil, 2020.

Inquerito CAP	Sugestões dos Juízes Especialistas	Decisão
Questão 01	*J10: “se o público que responderá o questionário será somente de usuárias atuais de pílula, ou que já usaram em algum momento da vida, nem é necessário fazer a primeira pergunta”.*	Remover
Questão 02	*J01: “simplificar o segundo ponto”.*	Acatar
	*J04: “considerando o público, que geralmente não têm muitas instruções básicas, a utilização de termos técnicos ainda que relevantes, talvez não seja bem compreendido”.*	
Questão 03	*J01: “as pílulas anticoncepcionais possuem outros benefícios além de evitar a gravidez?”*	Acatar
	*J09: “As pílulas anticoncepcionais possuem outros benefícios além da prevenção da gravidez?”*	
	*J1: “utilizar uma linguagem que se adeque a todos os níveis de escolaridade das entrevistadas”.*	
	*J12: “acredito que se a próxima pergunta não viesse elencado os benefícios elas poderiam não compreender o que você quer perguntar. (...) sugiro uma nova reescrita, por exemplo: Além de evitar a gravidez, as pílulas anticoncepcionais possuem benefício (s). O termo anticoncepcional é mais conhecido por elas do que o termo contraceptivo”.*	
Questão 04	*J01: “sugiro retirar “atua como coadjuvante” e colocar “auxilia” (...) Sugiro retirar “enxaqueca com aura” e deixar apenas “enxaqueca”, a condição “com aura” pode limitar o grau de resposta. Se possível, o termo “Doenças benignas” deveria ser simplificado”.*	Acatar
	*J04: “considerando o público, que geralmente não têm muitas instruções básicas, a utilização de termos técnicos ainda que relevantes, talvez não seja bem compreendido, mas em sua maioria estão de fácil compreensão, salvos os termos só são utilizados por profission- ais de saúde””*	
	*J11: “uma linguagem que se adeque a todos os níveis de escolaridade das entrevistadas”. Ademais, o J06 pontuou o seguinte: “Sugiro que esclareça os termos usados (ex:aura). No item protege contra doença benignas da mama. Colocar entre os parênteses (exemplos: lipoma, fibroadenoma, AFBM”.*	
Questão 07	*J01: “Sugiro definir o uso de pílula, e não “comprimido” (e para todo o instrumento). Sugiro retirar “sangramento menstrual” e colocar “primeiro dia da menstruação”.*	Acatar
Questão 08	*J01: “sugiro mudar a pergunta para “Como se deve tomar a pílula anticoncepcional?” (...) Sugiro troca de “ingerir um comprimido” por “ingerir uma pílula”. Sugiro incluir “Se a cartela for de 28 ou 35 PÍLULAS, não FAZER pausa”. A mesma coisa para os demais pontos. (...) “E sobre pausa no final da cartela” - sugiro trocar por “Se a cartela for de 21 pílulas, fazer pausa...”.*	Acatar
	*J08: “Sugiro rever a redação, pois o item 2 acrescenta a informação sobre o esquecimento da pílula não abordado no item 1. Além disso, o esquecimento de uma pílula, bem a ingestão no mesmo horário, estão diretamente relacionados à eficácia do método, mas não quer dizer que é um erro inaceitável, pois mesmo tomando em horários alternados e esquecendo uma pílula, o risco de gestação é menor comparando com a mulher que não toma de jeito nenhum e tem relações desprotegidas”.*	
Questão 09	*J01: “Sugiro começar por “Tomar duas pílulas (a do dia anterior e a do dia atual), imediatamente, no horário que lembrar. Após isso, tomar as demais pílulas da cartela, regularmente, uma a cada dia. (...) Sugiro retirar “deve” e iniciar com o verbo “Tomar”. (...) sugiro consertar a frase. Ex.: “Não tomar a pílula e usar apenas camisinha (preservativo). Iniciar nova cartela no início da próxima menstruação”*	Acatar
	*J05: “1- ( ) Deve tomar as duas pílulas (apenas colocar a palavra pílula no plural para devida concordância).”; e J06 - “Deve tomar as duas pílulas (correção ortográfica)”.*	
Questão 10	*J01: “Sugiro rever frase “contar quantas pílulas restam na cartela (...)”, há 3 ideias numa única frase. Melhor quebrá-la em 3. Ex.: “Contar quantas pílulas restam na cartela. Caso restem sete ou mais pílulas, tomá-las como de costume. No entanto, caso restem menos de sete pílulas, tomá-las e iniciar uma nova cartela um dia após ingerir a última pílula da cartela anterior.” (...) Sugiro trocar “deve pular” por “Ignorar pílulas esquecidas e tomar as demais, usar camisinha (preservativo) ou evitar relações sexuais durante sete dias”. Rever se não é possível “quebrar” a oração em 2 ou 3”.*	Acatar
Questão 11	*J01: “Sugiro retirar “mecanismos de ação” e colocar “Como agem as pílulas anticoncepcionais?” (...) Sugiro retirar “ascensão dos espermato- zoides” e colocar apenas “dificultando a penetração dos espermatozoides”. (...) Sugiro colocar apenas “Reduzem a contração das tubas uterinas” (se possível, explicar o que são). (...) sugiro “Matam os espermatozoides” P5 - se possível, explicar o que é ovulação”.*	Acatar
	*J07: “Utilizar uma linguagem adequada nas alternativas”.*	
Questão 12	*J01: “Rever a pergunta. Sugiro “Quais são os outros efeitos das pílulas anticoncepcionais? Marque aquele (s) que não impede(m) a continuação do uso. 1 - Aumento de sensibilidade nos seios. 9 - Explicar “baixo ventre”. Sugiro rever ordem de colocação dos pontos. Reveja a possibilidade de colocar o ponto 2 abaixo do 1, o três abaixo do 2, e assim por diante. A disposição atual pode confundir o entrevistador”.*	Acatar
	*J10: “Qual (is) o (s) principal (is) efeito (s) secundário (s) ou complicações do uso das pílulas anticoncepcionais? Colocando como alternativas de resposta todas as que estão na pergunta 12 e todas que estão na pergunta 13. Entre esses efeitos ou complicações que você assinalou acima, quais não impedem a continuação do uso? 14. Entre esses efeitos ou complicações que você assinalou acima, que impedem a continuação do uso?”*	
Questão 13	*J10: “Colocando como alternativas de resposta todas as que estão na pergunta 12 e todas que estão na pergunta 13”.*	Remover
Questão 14	*J01: “Sugiro colocar “Marque/Quais as principais contraindicações para o uso dos anticoncepcionais”. Inserir “pílula” antes do nome “a. combinada”. Sugiro trocar “tabagistas” por “mulheres que fumam e tem mais de 35 anos”. Rever “hipertensão arterial”. Em outras questões, foi colocado “pressão alta”. O mesmo para “enxaqueca com aura”.*	Acatar
	*J04: “Considerando o público, que geralmente não têm muitas instruções básicas, a utilização de termos técnicos ainda que relevantes, talvez não seja bem compreendido”*	
	*J09: “Sugestão: Quais mulheres não podem usar as pílulas anticoncepcionais”.*	
Questão 15	*J01: “Essa é uma pergunta muito difícil para se avaliar. Apesar de importante o conhecimento, quem faz a avaliação de interação medicamentosa é um profissional da saúde”.*	Manter
	*J06:” as pílulas anticoncepcionais podem interagir com outros medicamentos? Este questionamento é extremamente relevante tendo em vista que muitos profissionais não se atentam para estas interações, principalmente a Rifampicina (nos casos de tuberculose e hanseníase)”.*	
Questão 16	*J09: “Sugestão: Por que você usaria a pílula anticoncepcional?”*	Acatar
Questão 17	*J05: “Seria conveniente dividir essa pergunta em duas, pois existem mulheres que tomam as pílulas todos os dias, no entanto não no mesmo horário”.*	Acatar
Questão 18	*J01: “Rever “comprimido” / “sangramento menstrual”.*	Acatar
Questão 20	*J01: “Sugestão: “1 - “Tomar as duas pílulas”. Rever o termo “pílula regular”. Sugiro “pílula do dia”.*	Acatar
	*J09: “Sugestão: O que você faria se esquecesse de tomar a pílula anticoncepcional?”*	
Questão 21	*J01: “Sugiro rever o texto. Ex.: A questão pergunta: “O que (você) faria?” Então, sugiro colocar o texto de acordo com a pergunta: (eu) “Tomaria uma pílula imediatamente”. “Usaria camisinha ou evitaria relações sexuais durante sete dias”. “Contaria quantas pílulas”. - É a atitude da pessoa investigada, então deve ser na primeira pessoa. Rever os pontos 1, 2 e 3 e “quebrar” as frases em 2 ou 3”.;*	Acatar
	*J05: “ver o tempo verbal das opções 1, 2 e 3”.*	
	*J09: “Sugestão: O que você faria se esquecesse de tomas duas ou mais pílulas anticoncepcionais?”*	
Questão 22	*J01: “Sugiro: “Se a cartela fosse de 21 PÍLULAS, faria UMA pausa de sete dias e iniciaria UMA nova cartela no oitavo dia. CASO fosse de 22 PÍLULAS, faria UMA pausa de seis dias e iniciaria UMA nova cartela no sétimo dia. CASO fosse de 28 ou 35 PÍLULAS, não faria pausa, e observaria a recomendação da bula.” 2 - Sugiro rever “comprimido” e trocar por “pílulas”.*	Acatar
Questão 23	*J01: “Os motivos poderiam ser outros também. Ex.: “Sim, para tirar dúvidas” ou “Sim, mas apenas para buscar outra cartela”. Os autores poderiam colocar um espaço para o motivo do “não” e colocar um outro ponto com “Sim, mas por outro motivo. Qual?”*	Acatar
Questão 25	*J09: “Sugestão: Você procurou algum profissional da saúde antes de começar a usar a pílula anticoncepcional?”*	Acatar
Questão 26	*J09: “Sugestão: Se SIM, na questão anterior. Alguma vez você procurou algum profissional da saúde depois que começou a usar pílula anticoncepcional?”*	Acatar
Questão 27	*J09: “Sugestão: Se SIM, na questão anterior. Alguma vez você procurou algum profissional da saúde depois que começou a usar pílula anticoncepcional?”*	Acatar
Questão 32	*J01: “Rever “primeiro dia de sangramento menstrual” e o português. Ex.: corrigir o “(...), a, no máximo, o quinto dia, da menstruação”. J09: “Sugestão: Você já esqueceu alguma vez de tomar a pílula anticoncepcional durante o mês?”.*	Acatar
Questão 34	*J01: “Faz ou fez? Sugiro trocar “Deixei as pílulas esquecidas para trás” por “Não tomei as pílulas que esqueci, tomei apenas as que lembrei, e comecei a usa camisinha e a evitar relações sexuais durante sete dias. Costumo iniciar nova cartela um dia após a última pílula.”.*	Acatar
	*J05 - “Ver também o tempo verbal das demais opções e o texto das perguntas que parece estarem incompletas.”.*	
	*J09 - “Pergunta: Se sim na questão 31 e a resposta foi duas ou mais. Quando você esqueceu de tomar duas ou mais pílulas, o que você faz?* *Sugestão: acredito que é a questão 32.*	

Fonte: Dados da pesquisa, 2020.

No Gráfico 1, é revelado o IVC total do inquérito CAP por juízes, obtido por meio do somatório dos valores 4 ou 5 dados por eles em cada item (clareza, pertinência e rele- vância) de cada questão, dividido pelo quantitativo de itens avaliados. Na avalição de 10 (83,3%) juízes, o inqué- rito CAP obteve valores de IVC igual ou superior a 0,8 ou 80,0%. Quanto ao IVC total do inquérito, que se deu por meio do somatório do IVC obtido na avaliação de cada juiz, dividido pelo total de juízes especialistas participan- tes da pesquisa, foi de IVC total de 0,86 (86,0%).

**Gráfico 1:**
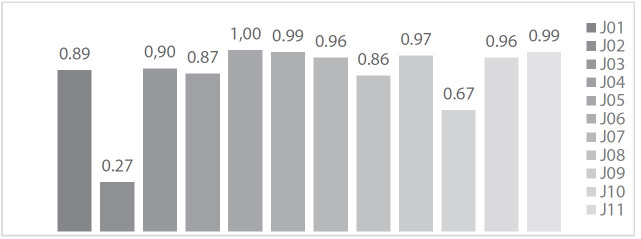
Apresentação de Índice de Validade de Conteúdo total por juiz especialista participante do estudo. Floriano, Piauí, Brasil, 2020.

Fonte: Dados da pesquisa, 2020.

É primordial destacar que, depois da realização de todos os ajustes no Inquérito CAP sobre o uso do anticoncepcional oral, o instrumento passou a conter as 35 questões, distribuídas da seguinte forma: 15 sobre os conhecimentos; 09, acerca das atitudes; e 11, da prática.

## Discussão

A questão 01 do inquérito (“Você sabe ou já ouviu falar sobre o que é a pílula anticoncepcional?”) obteve IVC total de 0,8. Embora fosse clara, no item relevância e pertinência, ela apresentou um índice menor do que aquele considerado como padrão. Outros inquéritos CAP, um sobre uso do preservativo masculino e outro acerca do exame Papanicolau, possuíam como primeira questão no tópico sobre conhecimentos uma pergunta similar à apresentada(31),(32).

Esses dois inquéritos tinham perguntas que indagavam se o participante sabe ou já ouviu falar acerca do assunto abordado, visto que o uso daquele método ou a realização do exame citado não eram prerrequisitos para o público-alvo que responderia ao instrumento. Já o inquérito do presente estudo deverá, futuramente, ser respondido apenas por mulheres que já utilizaram ou estarão em uso de contraceptivo hormonal oral, diante disso, os pesquisadores decidiram por seguir a sugestão de um dos juízes sobre remover a questão.

A questão 15 (“As pílulas anticoncepcionais podem interagir com outros medicamentos?”) obteve IVC total de 0,77. Este resultado levou à análise minuciosa da pergunta. Destaca-se que as sugestões dos juízes foram dicotômicas, sendo assim, os pesquisadores decidiram por manter a pergunta tendo em vista a relevância dela, pois é imprescindível que as usuárias tenham conhecimento e entendimento das possíveis interações medicamentosas, que podem resultar em risco à saúde, bem como, em diminuir a eficácia contraceptiva do método e que, enquanto estiverem em tratamento, a utilização de outro método de suporte deverá acontecer

Quando os anticoncepcionais hormonais orais são ingeridos, o estrogênio e a progesterona são absorvidos pela mucosa intestinal lançados na corrente sanguínea e conduzidos para o fígado (ciclo entero-hepático). Porém, quando ocorre a utilização de um antibiótico bactérias da flora intestinal são eliminadas, as quais são responsáveis pela hidrólise dos conjugados estrogênios, assim, o ciclo êntero-hepático do estrógeno é diminuído, tendo uma redução dos níveis plasmáticos de estrógeno ativo(33).

Desse modo, os medicamentos que apresentam maiores interações com os anticoncepcionais hormonais orais são os antibióticos de largo espectro. Em 1985, foi comprovado cientificamente que a rifampicina, antibiótico para o tratamento de tuberculose, teve alta interferência no metabolismo dos contraceptivos orais. Além dele, a amoxicilina, eritromicina, penicilina e tetraciclinas também apresentam interação com esse método contraceptivo. Ademais, os anticonvulsivantes (fenobarbital, fenitoínas, primidona e carbamazepina) e antifúngicos (griseofulvina) agem nos níveis plasmáticos diminuindo assim a eficácia contraceptiva(4),(34).

Frente a isso, reforça-se a importância de que o conhecimento, a atitude e a prática das mulheres quanto ao uso de anticoncepcional hormonal oral mediante utilização de outro medicamento sejam investigados, a fim de que, quando detectado que esses são inadequados, os profissionais de saúde possam traças estratégias mais eficazes de educação em saúde sobre a interação medicamentosa desse método contraceptivo com outros medicamentos, de modo a prevenir uma gravidez não planejada.

Quanto às demais questões do inquérito CAP que foram revisadas, afirma-se de forma sintética que as recomendações dos juízes especialistas foram no sentido de tornar perguntas e respostas mais compreensíveis pelo público-alvo, sem ser pontuado erro no conteúdo do tema ora abordado, como: substituição de termos técnicos; padronização do termo pílula em todo o instrumento; redução da quantidade de palavras em perguntas e/ou respostas; e/ou transformação de uma questão em duas.

Do mesmo modo, estudo metodológico, que teve como objetivo elaborar uma caderneta de acompanhamento e orientação em saúde sobre a doença falciforme para familiares de crianças com essa enfermidade e realizar a sua validação, revelou que algumas das principais contribuições dos juízes especialistas estiveram relacionadas à substituição e ao acréscimo de alguns termos, entre outas, visando facilitar a compreensão do público-alvo quanto as orientações apresentadas(35).

Semelhantemente, em pesquisa, que objetivou descrever o processo de elaboração e validação de conteúdo acerca do uso do preservativo para a aplicação na educação em saúde, no âmbito da aprendizagem baseada em problemas, também foi identificado que alguns avaliadores sugeriram alterações para tornar o texto compreensivo e mais claro também(36).

Isso se justifica pelo fato de que a linguagem das informações encontradas na literatura deve ser transformada, tornando-as acessíveis às camadas sociais predominantes no Sistema Único de Saúde brasileiro, independentemente do grau de instrução das pessoas, visto que, muitas vezes, se percebe a utilização de linguagem técnica, que só os profissionais da área compreendem(13),(37).

Para finalizar é importante destacar que o inquérito CAP construído e validado é claro, pertinente e relevante para aquilo que se destina e dele se espera, pois obteve IVC total de 0,86 (86,0%). Em face disso, revela-se que o objetivo deste estudo foi atingido. Estudos semelhantes não foram identificados, porém, identificou-se um estudo que elaborou e utilizou com o público-alvo um CAP de uso consistente de anticoncepcionais orais combinados, entretanto, não efetuou a etapa de validação(38).

Outros instrumentos para avaliar conhecimentos, atitudes e/ou prática, mas destinados a diversos métodos anticoncepcionais, também foram construídos e validados satisfatoriamente por juízes especialistas, profissionais de saúde e/ou pacientes, como: inquérito Conhecimento, Atitude e Prática sobre o método dos dias fixos(39); e instrumento Avaliação do Conhecimento em Contracepção (em inglês: *Contraceptive Knowledge Assessment* - CKA)(40).

Tal achado desvela, pois, a contribuição desta pesquisa para o meio acadêmico e assistencial por haver levado à construção e validação de um instrumento que permitirá a avaliação, bem como a identificação de lacunas, do conhecimento, das atitudes e da prática de mulheres quanto ao uso de anticoncepcionais hormonais, sejam eles combinados ou isolados.

## Conclusões

Como observado, o instrumento construído e validado obteve um IVC superior ao parâmetro adotado na pesquisa, desse modo, conclui-se que o “Inquérito Conhecimentos, Atitudes e Prática sobre o Uso de Anticoncepcional Hormonal Oral” é um instrumento válido para ser aplicado com a finalidade de obter um diagnóstico situacional dos níveis de conhecimentos, de atitudes e prática de uma dada população que faz uso de contraceptivos orais, impactando positivamente nas ações de planejamento familiar ou reprodutivo.

Ademais, sugere-se a elaboração de um procedimento operacional padrão (POP), para auxiliar os futuros colaboradores durante a aplicação do instrumento com o público-alvo, visto que algumas questões possuem mais de uma resposta correta, bem como para sanar possíveis dúvidas das participantes sobre possíveis termos.

Todavia, há que se destacar aqui as limitações do presente estudo: o inquérito não foi validado pelo público-alvo; e não houve realizado de um teste de legibilidade das questões. Frente a isso, recomenda-se a realização de estudos que validem o instrumento com mulheres em idade fértil e que fizeram ou fazem uso de anticoncepcionais hormonais orais, bem como se aplique o teste de legibilidade a fim de observar se a leitura e, consequentemente a compreensão, das perguntas e respostas são acessíveis à população. Com isso, ter-se-á um instrumento eficaz para o que se pretende, o qual contribuirá para que os profissionais de saúde, em especial os enfermeiros, no momento do planejamento familiar, tomem decisões seguras.
